# Prevalence and determinants of the use of self-tests by members of the public: a mixed methods study

**DOI:** 10.1186/1471-2458-6-193

**Published:** 2006-07-25

**Authors:** Angela Ryan, Sheila Greenfield, Sue Wilson

**Affiliations:** 1 Department of Primary Care and General Practice, The University of Birmingham, Edgbaston, Birmingham B15 2TT, UK

## Abstract

**Background:**

Self-tests can be used by members of the public to diagnose conditions without involving a doctor, nurse or other health professional. As technologies to design and manufacture diagnostic tests have developed, a range of self-tests have become available to the public to buy over-the-counter and via the Internet. This study aims to describe how many people have used self-tests and identify factors associated with their use.

**Methods:**

A postal questionnaire will elicit basic information, including sociodemographic characteristics, and whether the person has used or would use specified self-tests. Consent will be sought to recontact people who want to participate further in the study, and interviews and focus groups will be used to develop hypotheses about factors associated with self-test use. These hypotheses will be tested in a case-control study. An in-depth questionnaire will be developed incorporating the identified factors. This will be sent to: people who have used a self-test (cases); people who have not used a self-test but would use one in the future (controls); and people who have not used and would not use a self-test (controls). Logistic regression analysis will be used to establish which factors are associated with self-test use.

**Discussion:**

Self-tests do have potential benefits, for example privacy and convenience, but also potential harms, for example delay seeking treatment after a true negative result when the symptoms are actually due to another condition. It is anticipated that the outcomes from this study will include recommendations about how to improve the appropriate use of self-tests and existing health services, as well as information to prepare health professionals for patients who have used self-tests.

## Background

Members of the public have become more involved in their own care. They use pregnancy tests and tests to monitor diagnosed conditions, such as diabetes mellitus [1], and self blood pressure measurement is popular [2]. Initiatives such as NHS Direct and the need to control costs have contributed to the development of self-care [3,4], as highlighted by the increasing scope for self-medication [5]. While this has been happening, technologies to design and manufacture tests that can be used in the home have advanced. As a result, a wider range of diagnostic and screening tests have become available to the general public [6]. These include tests for chlamydia, prostate specific antigen and faecal occult blood. Results are available immediately or after sending a sample to a laboratory, but contact with a doctor, nurse or other health professional is not necessary. These "self-tests" are likely to become even more easily available and widely used as the Internet continues to reduce physical and intellectual barriers.

People may use self-tests because of benefits of being tested outside a conventional medical setting. For example, people who would not visit a health professional may screen themselves because it is more convenient and private [7]. There are, however, also potential harms from being tested in this way. For example, a person who receives results without an interpretation of the whole picture, including signs and symptoms, could think he/she has a disease inappropriately, or a person could delay seeking treatment after a true negative result when his/her symptoms are actually due to another condition. There will also be false positive and false negative results. Self-tests are used without a formal independent assessment of these harms and benefits. Even if members of the public use tests that have been assessed as being beneficial when used during conventional screening programmes, their use is outside those quality assured programmes. Possible doubts associated with such testing include test accuracy, that people who are most at risk may not use self-tests, and that people with positive results may not actually get treated [8].

Many general practitioners feel that their workload has grown due to the move towards a primary-care-led NHS, that their prescribing behaviour is affected by patient demand, and that the number of demanding patients has risen [9-11]. An expansion of the use of self-tests may also exacerbate this perceived increase in demand and workload as people seek an explanation of results or further investigation.

Despite the potential impact of self-tests, the extent of their use is not known. Other than market research, a comprehensive literature review identified only one recent survey in the United Kingdom that asked participants about whether they had used home testing kits, and this was part of a study on attitudes to genetic testing [12]. There is also an absence of studies about why people use self-tests and perceived and actual harms and benefits.

### Pilot work

During September and October 2004, we sent questionnaires and prepaid envelopes to 380 addresses randomly selected from the Birmingham South West 2004/05 residential telephone directory. The questionnaire asked whether the respondent had used or would use specified self-tests, with room to add any not listed. We wanted to maximise responses from men and women because some self-tests, for example for prostate disorders, would only be used by one sex. We, therefore, sent two questionnaires to each address with a request for the addressee to give the additional questionnaire to any other adult living at the same address. The questionnaire asked whether the respondent lived alone to allow us to estimate the denominator.

Three questionnaires were returned because the addressee had died or moved, and 184 completed questionnaires were received from the remaining 377 households. The denominator for the response rate (n = 697) was estimated as the 57 respondents who reported that they lived alone plus two adults at each of the remaining 320 addresses, and the response rate was 26%. Excluding 22 people who had only used a pregnancy test, 28 (15%) respondents said they had used a self-test. Respondents most commonly reported using a test for diabetes (n = 18), but they also reported using tests for cholesterol, infertility, urinary infection, haematuria, prostate specific antigen and HIV-infection. Sixty two percent (n = 96) of the 154 respondents who had not used a self-test other than a pregnancy test said they would use one in the future.

### Study aims

The primary aims of this study are to describe the prevalence of the use of self-tests by members of the public to diagnose or screen for conditions without the involvement of a doctor, nurse or other health professional, and to determine factors that are associated with their use.

## Methods

### Study design

Mixed methods two-stage study with (1) an initial survey comprising a postal questionnaire, interviews and focus groups, followed by (2) an embedded case-control study.

### Selection criteria

Adults aged 18 years or older randomly selected from participating general practices will be asked to complete the initial questionnaire. Practices will be selected to reflect the diversity of the population based on deprivation indicators, population density and ethnicity.

### Exclusion criteria

The initial questionnaire will not be sent to people who the general practitioner feels that it would be inappropriate to approach, for example people with a severe mental illness, terminal illness or recent bereavement.

### Methods of data collection

The initial survey involves a postal questionnaire that will elicit basic information about sociodemographic characteristics (e.g. age, sex, ethnic group, employment status), health status, and whether the person has used or would use self-tests that have been identified as available from a search of the Internet. The questionnaire will be designed using lessons learnt from the pilot survey and will be piloted with a small sample of the target population before being widely distributed. A cover letter will outline the study in lay terms and ask people to complete the questionnaire and return it in the enclosed prepaid envelope. The letter will be on headed notepaper from the person's general practice and signed by a partner at the practice. People will be given the option of returning a blank questionnaire to indicate that they do not want to take part. One reminder will be sent to non-responders. This first questionnaire will seek consent for recontacting people about further participation in the study.

Interviews are suitable for gaining an in-depth understanding of personal experience and perspectives [13], and we will use interviews to investigate factors that may have influenced whether people used self-tests. Focus groups can be used to pilot ideas and questions [14,15], and we will conduct focus groups with people who have not used self-tests to reflect on the general applicability of factors identified in the interviews. People who consented to be recontacted about talking with a researcher will be sent an information leaflet and a reply slip to indicate if they would like to take part in an interview or focus group with a prepaid envelope to return the slip. To put people at ease and increase the likelihood of interaction, focus groups will include people of the same sex and similar ages. A semi-structured topic guide will be used for the interviews, and the topic guide for the focus groups will be based on factors identified during the interviews.

Results from the postal questionnaire, interviews and focus groups will be used to develop hypotheses about factors that may influence use of self-tests other than pregnancy tests: pregnancy tests will be excluded as women have used them for some time and their use is probably now expected by doctors. These hypotheses will then be tested in a case-control study. An in-depth questionnaire will be developed incorporating the factors of interest. The questionnaire will be piloted with a small number of people from the target groups before it is sent to: cases who have used a self-test; controls who have not used a self-test but would use one in the future; and controls who have not used a self-test and would not use one in the future. This staging of questionnaires has been successfully used in other studies and is believed to generate a better response rate then sending a longer initial questionnaire [16]. A cover letter will outline the study in lay terms and ask people to consider completing the questionnaire and returning it using the enclosed prepaid envelope. People will be given the option of returning a blank questionnaire to indicate that they do not want to take part. One reminder will be sent to people who do not return a questionnaire.

### Justification of sample size

As pregnancy tests will be excluded, self-test in this and the next section refers to tests other than pregnancy tests. Conservatively assuming that 10% of people have used a self-test, a sample of 4200 people will allow estimation of the prevalence of the use of self-tests with at least +/-1% precision and 95% confidence. Based on a response rate of 40%, which is less than other large prevalence surveys [16], the questionnaire will be sent to 10500 people. Assuming an average list size of 4500 people, 75% of whom are 18 years or older [17], and 5% of whom meet the exclusion criteria, it would be sufficient to recruit four general practices, but up to eight will be recruited to reflect diversity and increase generalisability.

It is assumed that 75% (n = 315) of the 420 respondents who have used a self-test and 50% (n = 1890) of the 3780 respondents who have not used a self-test will agree to be recontacted. Purposive sampling will be used to select people for interviews and focus groups [18]. For the interviews, we will select men and women of different ages (younger and older) who have used self-tests for different conditions (cancers, other chronic conditions, sexually transmitted infections, other acute infections or conditions). The ideal size for a focus group is between four and eight people [15], and we anticipate that each group will include six people who have not used self-tests. To facilitate sharing of views [14], we will hold groups with people of the same sex and similar ages. To allow loose matching by age (younger or older) and sex (male or female), up to four groups will be held. We anticipate, therefore, that interviews and focus groups will involve up to about 24 self-test users and 24 non-users [19].

Assuming that 75% of people who have used a self-test and 50% of people who have not used a self-test respond to the invitation to take part in an interview or focus group, we will need to approach 32 people who have used a self-test and 48 people who have not used a self-test to take part in this part of the study. This will leave 283 self-test users and 1842 non-users who have agreed to be recontacted.

In the absence of data relating to self-testing, we used factors associated with self-care to calculate a likely sample size for the case-control study. A Spanish study found that self-medication was more prevalent among people who lived alone [20]. About 15% of adults aged 16 years or over live alone [21,22]. If the same proportion of people who have not used a self-test live alone, data from 207 cases and 207 controls should detect a doubling of the odds of living alone among people who have used a self-test with 80% power and 5% significance.

Assuming that 75% of the remaining 283 self-test users return the in-depth questionnaire, there will be data from 212 cases. The pilot survey suggests that 62% (n = 1148) of the remaining 1842 non-users would consider using a self-test in the future, whereas 38% (n = 694) would have no interest in using one. Assuming a response rate of 50%, the in-depth questionnaire will need to be sent to 424 people from each of these groups to generate 212 controls from each group.

### Methods of data analysis

The prevalence of the use of self-tests will be estimated after appropriate standardisation to the national population, and 95% confidence intervals will be calculated. We will compare sociodemographic characteristics of people who have used, who have not used, who would use, and who would not use self-tests.

Interviews and focus groups will be recorded and fully transcribed. The transcripts will be read, coded, indexed and categorised, facilitated by appropriate software. We will use grounded theory, that is we will identify analytical categories as they emerge from the data and use these categories to develop hypotheses about factors that may be associated with using self-tests [23]. The analysis will be iterative: hypotheses will be tested as they emerge using analytic induction [23], and we will amend the anticipated number of interviews and focus groups depending upon whether new issues continue to emerge. Respondent validation will be sought by inviting feedback from participants who will be sent a written summary of the interview or focus group.

The data from the second in-depth questionnaire will be fully investigated and described using univariate and bivariate comparisons of people who have and who have not used a self-test. Stepwise logistic regression will then be used to test the hypotheses developed during the interviews and focus groups and examined by the second questionnaire, and to establish those factors that are associated with using a self-test.

Self-tests can be grouped according to the disease area, for example for cancer or sexually transmitted infections. Secondary analyses will be conducted to generate hypotheses about whether particular determinants of self-test use vary by the type of test and, therefore, whether future research should be test-specific.

### Bias and confounding

We aim to maximise compliance and minimise selection bias by keeping the demands on people to a minimum, but we will compare the characteristics of responders and non-responders and standardise the results to the national population. People who have participated in interviews or focus groups will be excluded from the case-control study as their responses could be affected by the discussion. During the case-control study, it is anticipated that cases and controls will be matched by sex and age group because they may be confounders: possible determinants of self-test use, such as access to the Internet, may vary with age. The criteria for matching will, however, be finalised after further information is collected during the interviews and focus groups. As the first questionnaire may "educate" people and lead to self-test use, the second questionnaire will be analysed on the basis of reported self-test use when the first questionnaire was distributed. To minimise recall bias, respondents to the second questionnaire will be asked to report behaviours and experiences over the preceding year.

### Ethical approval

This study has been approved by Solihull Local Research Ethics Committee, reference 05/Q2706/13.

## Discussion

The pilot study suggests that some people are using self-tests. These findings are in line with a 1993 survey in which 18% of respondents said they would prefer self-testing to testing by a doctor [24]. A recent British Medical Association report highlighted, however, that ad hoc screening can put people at risk because of a lack of evidence underpinning tests and insufficient quality assurance and accompanying information [25]. Self-tests do have potential benefits, for example privacy and convenience, but also potential harms, for example distress caused by false positive results [26]. Other potential problems include extra pressure on primary health care professionals and NHS laboratories as people seek an explanation of results or further investigation [27]. Despite this, there is an absence of studies about self-tests. We think that important first steps are to describe the prevalence of the use of self-tests and to determine factors that are associated with using them.

## Abbreviations

NHS = National Health Service.

## Competing interests

The authors declare that they have no competing interests.

## Authors' contributions

Angela Ryan, Sue Wilson and Sheila Greenfield designed the pilot questionnaire survey, and Angela Ryan conducted the survey and analysed the data. Angela Ryan drafted the study protocol with input from all authors. All authors read and approved the final manuscript.

## Pre-publication history

The pre-publication history for this paper can be accessed here:


